# Genome-scale transcriptional activation by non-homologous end joining-mediated integration in *Yarrowia lipolytica*

**DOI:** 10.1186/s13068-024-02472-x

**Published:** 2024-02-15

**Authors:** Xiaoqin Liu, Jingyu Deng, Jinhong Zhang, Zhiyong Cui, Qingsheng Qi, Jin Hou

**Affiliations:** State Key Laboratory of Microbial Technology, Shandong University, Binhai Road 72, Qingdao, 266237 Shandong People’s Republic of China

**Keywords:** Gene regulatory library, Non-homologous end joining, Targets identification, *Yarrowia lipolytica*

## Abstract

**Background:**

Genome-scale screening can be applied to efficiently mine for unknown genes with phenotypes of interest or special functions. It is also useful to identify new targets for engineering desirable properties of cell factories.

**Results:**

Here, we designed a new approach for genome-scale transcription activation using non-homologous end joining (NHEJ)-mediated integration in *Yarrowia lipolytica*. We utilized this approach to screen for genes that, upon activation, confer phenotypes including improved acetic acid tolerance and xylose metabolism. The candidates were validated using gene overexpression, and functional changes including improved growth performance under multiple stressors and activated pentose metabolism were identified.

**Conclusions:**

This study provides a simple and effective approach to randomly activate endogenous genes and mine for key targets associated with phenotypes of interest. The specific gene targets identified here will be useful for cell factory construction and biorefining lignocellulose.

**Supplementary Information:**

The online version contains supplementary material available at 10.1186/s13068-024-02472-x.

## Background

Genome-scale screening is a powerful approach to identify gene targets related to specific phenotypes. It is also important to mine new targets for cell factory construction. For example, genome-scale screening can be applied to identify gene targets that improve the stress tolerance of microorganisms and the efficiency of substrate utilization or product synthesis, contributing to the improved robustness or chemical synthesis capability of cell factories.

Approaches such as yeast deletion sets, genome-scale CRISPR/CRISPRi, and RNAi have been successfully applied in the identification of dominant gene targets and the study of genetic regulatory networks [1–4]. These genetic strategies are mainly used to eliminate or inhibit gene function, but are unable to find positive genetic-phenotypic correlations. To screen for genes with increased expression levels, genome-scale transcriptional activation is necessary. Current approaches used frequently for genome-scale transcriptional activation are the construction of gene overexpression library or CRISPR activation (CRISPRa) library. For example, the cDNA overexpression libraries or genomic DNA overexpression libraries have been generated for gene overexpression [5, 6]. However, the construction of these libraries is a labor-intensive process. Recently, CRISPRa combined with pooled guide RNA libraries has offered significant advantages as a promising tool for genome-wide functional screening [5]. CRISPRa fuses nuclease-deficient Cas9 (dCas9) with a transcriptional activator to target transcriptional regulatory regions and activate gene expression [7]. However, its efficiency is still affected by the specificity and efficiency of sgRNA. In addition, the fusion of only one transcriptional effector often fails to achieve efficient transcriptional regulation, and its range may not be suitable for genome-scale screening [8–10]. Therefore, hybrid trivalent activator VP64-p65-Rta (VPR) and transcription signal amplification systems were developed to enhance the efficiency and specificity of gene activation [11, 12].

*Yarrowia lipolytica* is a non-conventional yeast with strong stress tolerance and wide substrate utilization spectrum. It is considered a promising industrial microorganism [13–15]. Functional annotation of the *Y. lipolytica* genome has been mainly inferred from homology with the genes of model yeast and other fungi, and many gene functions have not been verified [16, 17]. The poor functional understanding of the *Y. lipolytica* genome makes it challenging to construct efficient cell factories by rational design alone. Genome-scale screening and gene mining are powerful approaches for genetic analysis and identification of phenotype-related genes.

*Y. lipolytica* tends to repair DNA double-strand breaks (DSBs) by non-homologous end joining (NHEJ) [18, 19]. In our previous study, we established a novel approach for generating genome-scale insertional mutagenesis library using NHEJ-mediated integration [20]. It can generate both gene disruption and gene repression. By systematically analyzing the genomic distribution of NHEJ-mediated integration, we found that the integration showed high integration preference in transcriptional regulatory regions, especially promoter regions. Therefore, in this study, we redesigned NHEJ-mediated integration library construction strategy to generate gene regulatory library that could achieve gene activation. Using this approach, we identified the causal genes that can improve the tolerance of acetic acid and xylose metabolism, which properties are important for lignocellulose utilization. This approach of NHEJ-mediated integration for genome-scale gene activation is simple and does not rely on complex design, and has the potential to become a powerful genetic perturbation technology. The targets identified can be applied for constructing the cell factory that can use lignocellulose feedstock.

## Results and discussion

### Design NHEJ-mediated integration for gene activation

**Fig. 1:**
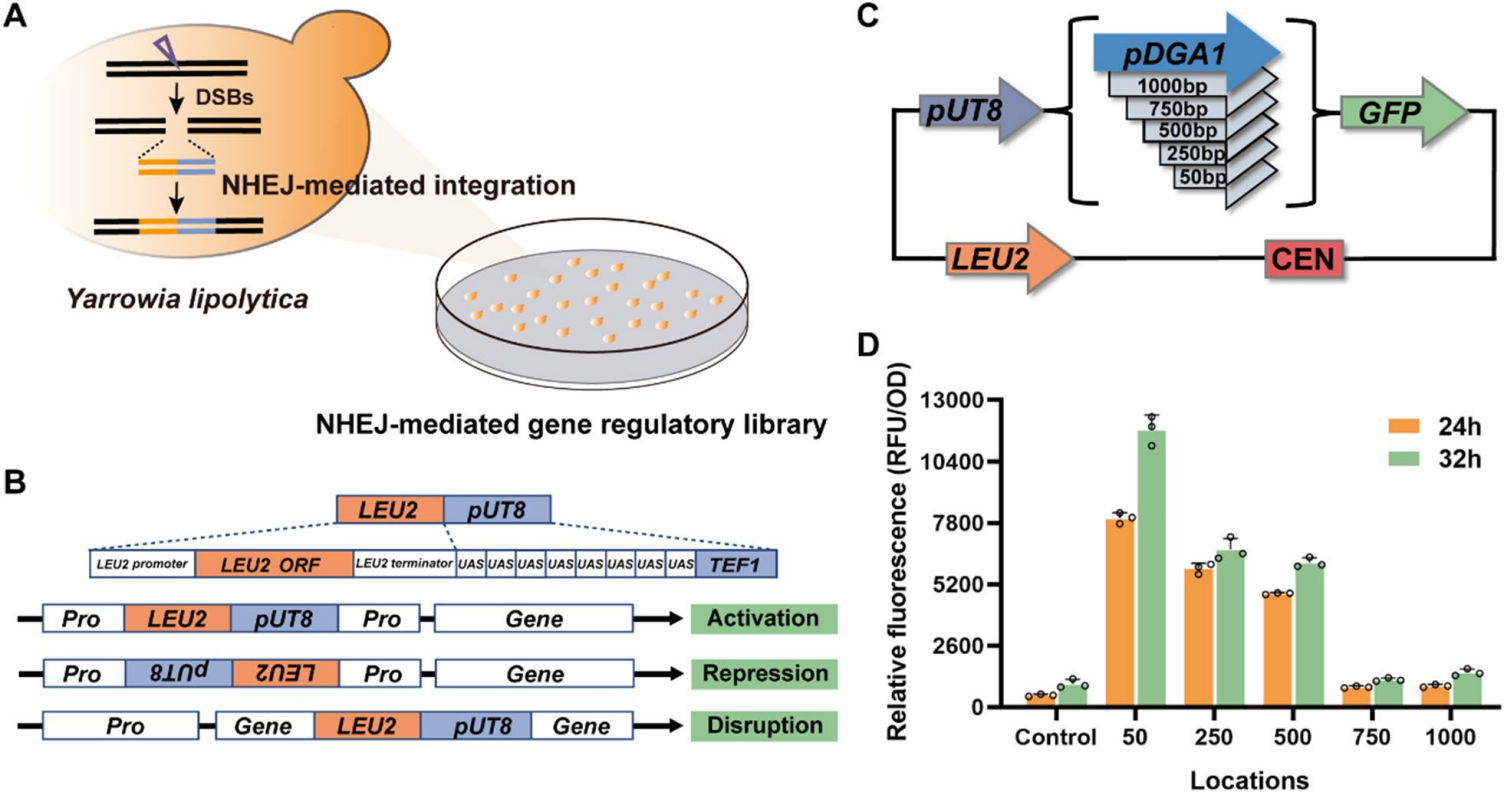
Feasibility validation of NHEJ-mediated gene regulatory library for gene activation. **A** Schematic diagram of constructing genome-scale gene regulatory library by NHEJ-mediated random integration. **B** Schematic diagram of the effect of different insertion positions of the *LEU2-UT8* fragment on gene expression. When the *LEU2-UT8* DNA fragment is inserted into the genomic promoter (Pro) region with the same 5’-3’ direction, it may activate gene expression. When it is inserted in the opposite direction, it may disrupt the function of the endogenous promoter, leading to the repression of downstream genes. Insertion within a gene will disrupt gene expression. **C** Schematic diagram of inserting the *UT8* promoter into different positions of the *DGA1* promoter to control *hrGFP* expression. **D** Effect of different positions of *UT8* promoter inserted into *DGA1* promoter on *hrGFP* expression

In our previous study, a genome-scale insertional mutagenesis library was constructed via NHEJ-mediated random integration of a marker gene and collection of > 10^6^ colonies. A map of the distribution of NHEJ-mediated integration revealed that a high integration frequency was achieved, with an average density of one integration per 22.4 base pairs (bp) throughout the genome. Although the integration occurred in both intergenic regions and open reading frames (ORFs), there was a preference for transcriptional regulatory regions, especially promoters [20]. The average integration frequency in 0 bp–250 bp upstream of ORF was 7.91-fold higher than that of the whole genome. Considering the importance of genome-scale gene activation in phenotype-to-genotype screening, we further tested the possibility of redesigning NHEJ-mediated integration to construct library for gene activation (Fig. 1A). A strong hybrid promoter (*UAS1B8-TEF1* [*UT8*]), comprising the *TEF1* core promoter and eight tandem upstream activating sequences of the *XPR2* promoter [21], was assembled with the *LEU2* expression cassette (*LEU2-UT8*) as the integration fragment (Fig. 1B). The strength of *UT8* is reportedly higher than that of the strong natural promoter of *Y. lipolytica*. To verify whether insertion of *UT8* in the promoter regions of the genome can increase the transcription of downstream gene, we tested its effects on the expression of downstream reporter gene encoding humanized *Renilla reniformis* green fluorescent protein (*hrGFP*), which was regulated by the endogenous weak promoter *DGA1* (Fig. 1C). As shown in Fig. 1D, the fluorescence intensity of *hrGFP* was low under independent regulation by the intact *DGA1* promoter (control). When the *UT8* promoter was assembled with the truncated *DGA1* promoter, leaving 50 bp, 250 bp or 500 bp at the 3’ end, respectively, the fluorescence intensity was increased to different degrees. With only 50 bp of the *DGA1* promoter at the 3’ end, the increase in fluorescence intensity was particularly strong (11-fold). In contrast, there was no significant increase in fluorescence intensity when 750 bp or 1000 bp of the *DGA1* promoter was left at the 3’ end. This result demonstrated that insertion of the *UT8* promoter in a promoter region could indeed raise the expression level of a downstream gene to varying degrees. In addition, the closer the *UT8* promoter to the gene coding sequence, the higher the increase in gene expression. Therefore, *UT8* promoter insertion in such regions is possible to activate gene expression.

To determine whether the insertion of *LEU2-UT8* is possible to construct a library that could achieve gene activation, we randomly integrated the promoterless hygromycin resistance gene (*HYG*) into the *Y. lipolytica* Po1f genome to obtain the strain Po1f-hyg. The strain did not grow on solid plates containing hygromycin (Additional file 1: Figure S1A). Next, *LEU2-UT8* fragment was transformed into Po1f-hyg to construct the insertional library. Approximately 2.0 × 10^5^ transformants were collected, some of which grew on hygromycin-resistant plates (Additional file 1: Figure S1B). Insertion of the *UT8* promoter near *HYG* in these transformants was confirmed by PCR (Additional file 1: Figure S1C). These results indicated that *LEU2-UT8* was inserted in the upstream regions of *HYG* gene and activated its expression.

Theoretically, when *LEU2-UT8* DNA fragments are inserted into the promoter region, the obtained insertional library can achieve gene activation or repression, depending on the direction and location of the inserted fragment (Fig. 1B).

### Identifying targets for improved acetic acid tolerance using NHEJ-mediated gene regulatory library

**Fig. 2:**
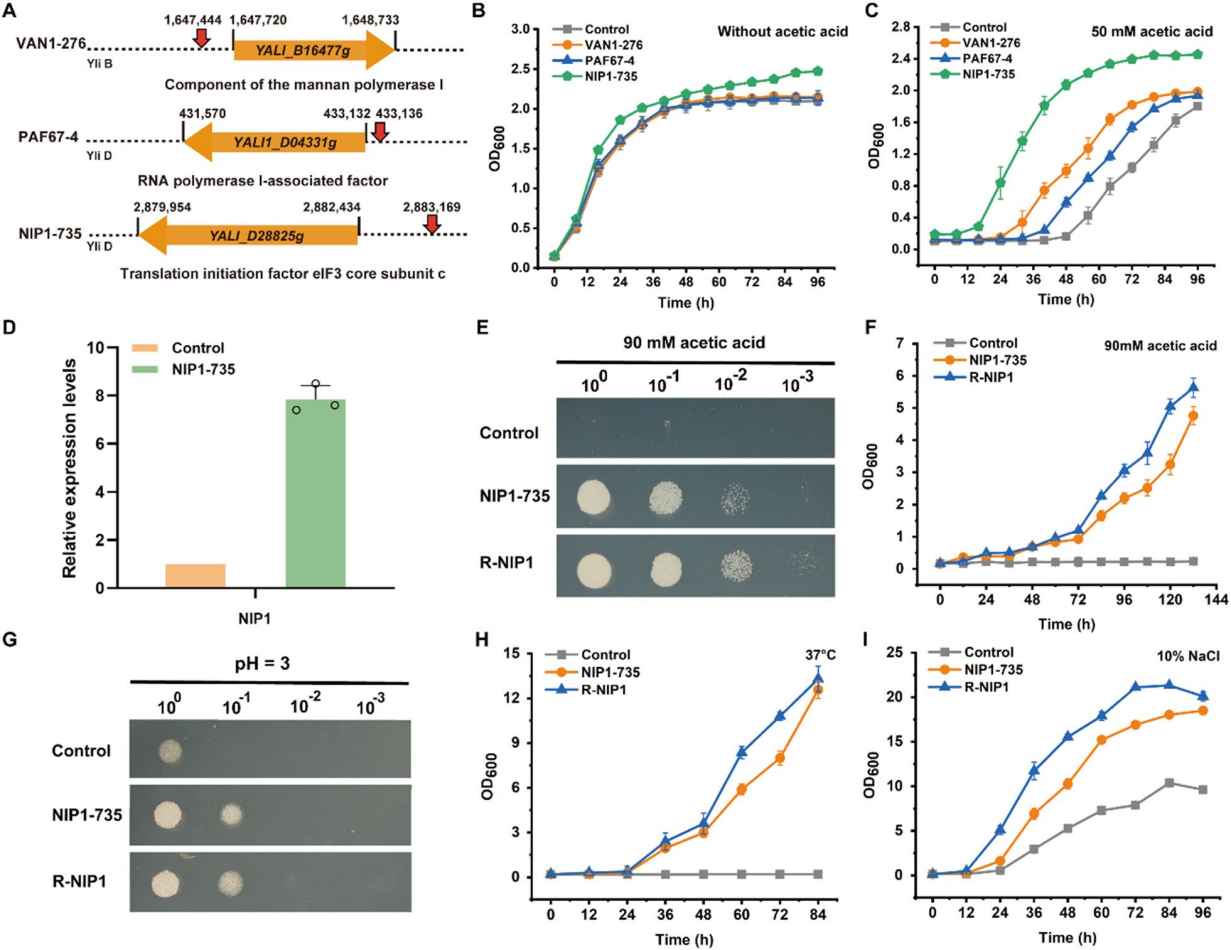
Identification of the mutants with improved acetic acid tolerance. **A** Insertion positions (red arrows) of the *LEU2-UT8* fragment in the mutant strains. **B**, **C** Growth curves of the *Y. lipolytica* control strain Po1f and the mutant strains without **B** and with **C** the addition of 50 mM acetic acid. **D** Relative transcription levels of *NIP1*. **E**, **F** Spot assay **E** and growth curves **F** of Po1f and the mutant strains under 90 mM acetic acid. **G** Spot assay of Po1f and the mutant strains under low pH. **H**, **I** Growth curves of Po1f and the mutant strains under high temperature **H** and 10% NaCl **I**. R-NIP1: *UT8* promoter-driven *NIP1* overexpression strain

In the first case study, we applied the NHEJ-mediated gene regulatory library to screen mutants that could improve acetic acid tolerance. Biorefining lignocellulose could provide a sustainable supply of fuels and chemicals. Acetic acid is one of inhibitors in lignocellulose hydrolysate. Therefore, identification of the causal genes that can improve acetic acid tolerance will facilitate lignocellulose utilization [22]. In the presence of 70 mM acetic acid, the growth of strain Po1f was inhibited (Additional file 1: Figure S2A). To obtain mutants with improved acetic acid tolerance, we constructed NHEJ-mediated gene regulatory library and performed enrichment screening of library mutants in medium supplemented with 70 mM acetic acid. Mutants with improved acetic acid tolerance were selected, and the location of *LEU2-UT8* fragment was analyzed and the targeted genes were identified. As shown in Fig. 2A and Additional file 1: Table S1, *LEU2-UT8* fragments were all inserted in the upstream region of the genes. These genes are *YALI1_B16477g*, the homologue of the *VAN1* of *S. cerevisiae*, *YALI1_D04331g*, a gene encoding RNA polymerase I-associated factor, and *YALI1_D28825g*, the homologue of *NIP1* of *S. cerevisiae*. Among them, we found that the *LEU2-UT8* fragment was inserted 735 bp upstream of the *NIP1* coding sequence in four mutants. As shown in Fig. 2B, C, the NIP1-735 strain showed the most significant improvement in acetic acid tolerance, and its growth was also better than the control in the absence of stress. NIP1 (YALI1_D28825g) is a subunit of the eukaryotic translation initiation factor 3 (eIF3) complex, and is involved in the assembly of the preinitiation complex and start codon selection [23–25]. eIF3 is an 800-kDa eukaryotic initiation factor composed of 13 subunits, which organizes the initiation factor and ribosome interactions required for productive translation [26]. NIP1 plays a critical role in 40S ribosome association [27]. It has been reported that NIP1-depleted cells have reduced protein synthesis rates and a defect in the translation initiation stage [23, 28].

We measured the transcription level of *NIP1* and found that the insertion of *UT8* upstream of the *NIP1* coding sequence increased its expression level by approximately eight-fold (Fig. 2D). Therefore, we overexpressed *NIP1* in the control strain to verify that the growth improvement was due to *NIP1* activation. The results showed that the *NIP1* overexpression strain, but not the control strain, grew under 90 mM acetic acid, confirming that *NIP1* overexpression increased acetic acid tolerance (Fig. 2E, F).

Interestingly, we found that *NIP1* activation improved cell growth regardless of the addition of acetic acid. To determine whether *NIP1* also protects cells from other stress conditions, we tested the tolerance of *NIP1* mutant strains to low pH, high temperature and high osmotic pressure. Compared with the control strains, the mutant strains also exhibited better growth under low pH, high temperature and high osmotic pressure (Fig. 2G, H and I). The above results indicated that overexpression of *NIP1* had a positive effect on the tolerance to multiple stressors, which will be very useful for robust cell factory construction. Reportedly, eIF3 may be associated with mechanisms that promote the synthesis of stress-related proteins [29]. Furthermore, overexpression of Int6, a component of human translation initiation factor eIF3, in *Schizosaccharomyces pomb*e resulted in multidrug resistance, and knockdown of Int6 resulted in slower growth of *S. pombe* cells [30]. We speculated that the activation of *NIP1* may increase the efficiency of protein synthesis, thereby contributing to improved cell growth. However, this possible mechanism requires further exploration.

### Identifying targets that activate xylose metabolism in *Y. lipolytica* using NHEJ-mediated gene regulatory library

Xylose is the second most abundant sugar in lignocellulosic hydrolysate, and the utilization of xylose can contribute to cellulose biorefinery [31]. Previous genome mining revealed that xylose metabolic pathway encoding genes including *ylXR*, *ylXDH* and *ylXK* exists in *Y. lipolytica* [32, 33]. However, we found that *Y. lipolytica* Po1f grew very poorly in the medium with xylose as the sole carbon source, indicating that the endogenous xylose metabolic pathway was not effective for growth [32, 34].

**Fig. 3:**
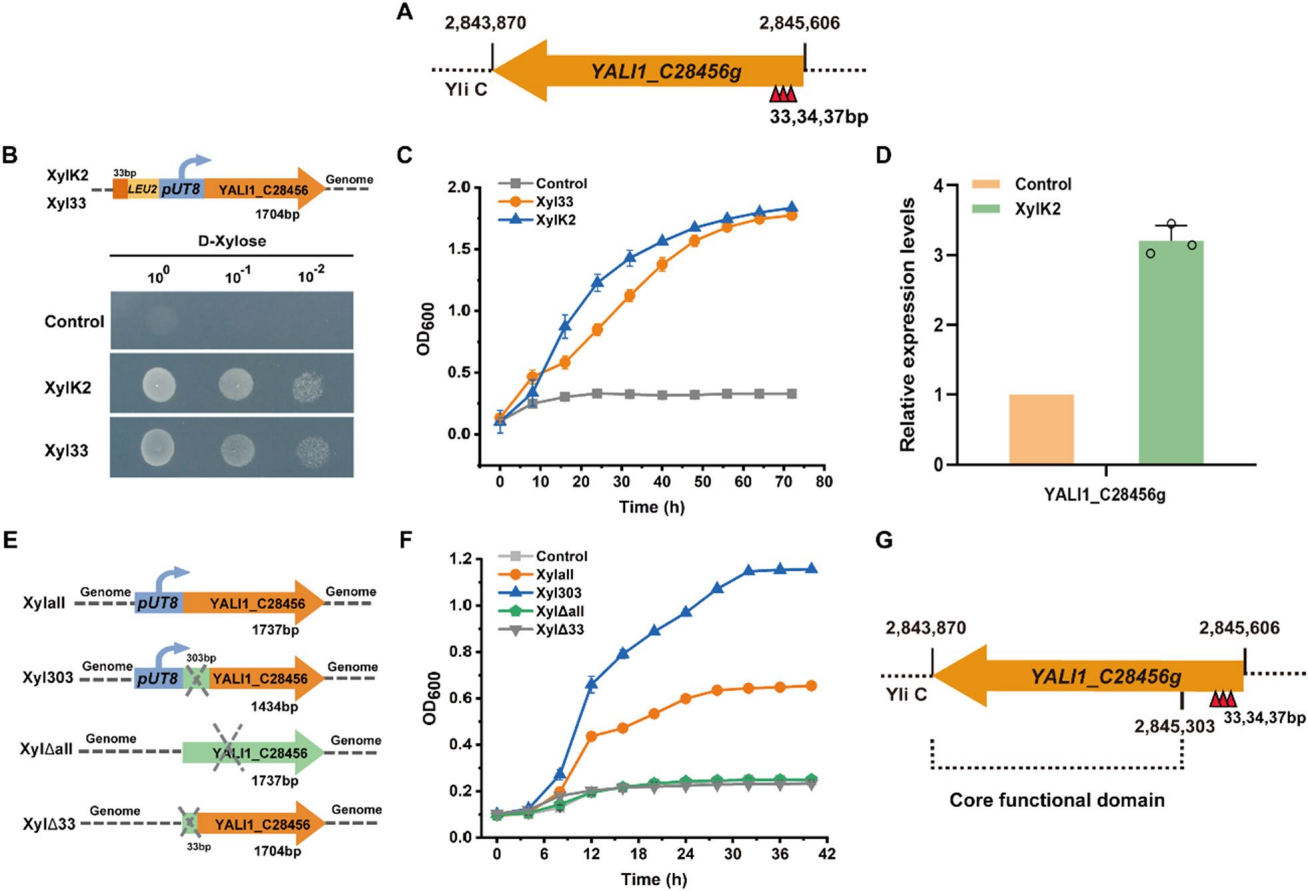
Identification of mutants with activated xylose metabolism in *Y. lipolytica*. **A** Insertion position (red triangle) of the *LEU2-UT8* fragment in the mutants. **B**, **C** Spot assay **B** and growth curves **C** of the control strain *Y. lipolytica* Po1f and the mutant strains with xylose as the sole carbon source. **D** Relative transcription levels of *YALI1_C28456g*. **E** Schematic diagram of genome modification of different mutants. **F** Growth curves of *YALI1_C28456g* overexpression and knockout mutant strains with xylose as the sole carbon source. **G** Redefinition of the *YALI1_C28456g* ORF. XylK2: library mutant strain with insertion of the *LEU2-UT8* fragment at the 33-bp position of *YALI1_C28456g*. Xylall: strain with *UT8* promoter-driven overexpression of *YALI1_C28456g*. Xyl303: strain with overexpression of *YALI1_C28456g* carrying a deletion of the 5′ 303 bp. XylΔall: *YALI1_C28456g* knockout strain. XylΔ33: strain with deletion of the 5′ 33 bp of *YALI1_C28456g*

To identify the targets that can activate xylose metabolism, we constructed a NHEJ-mediated gene regulatory library, and screened for mutants that could grow in the medium with xylose as the sole carbon source. The colonies with better growth were selected and the genomic insertion sites were analyzed. Interestingly, although the insertion positions were different, they all pointed to the same gene: *YALI1_C28456g*. According to the functional annotation of the *Y. lipolytica* genome in the National Center for Biotechnology Information database, the insertions were at the 33-bp/34-bp/37-bp region of the *YALI1_C28456g* ORF (Fig. 3A). Prior to this study, the function of YALI1_C28456g had not been analyzed. Sequence alignment revealed homology between YALI1_C28456g and the major facilitator superfamily (MFS) domain proteins (Additional file 1: Table S2). MFS is a large and diverse family of transporters, which facilitate the transport of various substances, such as amino acids, polypeptides, monosaccharides, and polysaccharides [35]. To further investigate this target, we inserted a *LEU2-UT8* fragment into the 33-bp position inside the gene (Xyl33) in strain Po1f for reverse engineering verification. Both the mutant strain XylK2 and the reverse-engineered strain Xyl33 grew better than the control strain by spot assay and growth characterization in the medium with xylose as the sole carbon source (Fig. 3B, C). This confirmed that *YALI1_C28456g* plays a key role in activating xylose metabolism in *Y. lipolytica*.

We expected the insertion of a large DNA fragment at the 33-bp/34-bp/37-bp coding region would inactivate *YALI1_C28456g*. However, measurement of the transcriptional level of *YALI1_C28456g* unexpectedly revealed that the *LEU2-UT8* insertion upregulated the transcription level (Fig. 3D). This phenomenon indicated that insertion of *LEU2-UT8* did not disrupt the gene, but rather activated it. Upon investigation of the YALI1_C28456g protein sequence, we found that, in addition to the first amino acid, the 102nd amino acid is also methionine. YALI1_C28456g contains 578 amino acids, and the region with homology to the MFS domain starts from the 102nd amino acid. This suggested that either the gene coding region from 0 to 303 bp is a transcriptional regulatory region, or the N-terminal 101 amino acids may not contain the core functional domain of the protein.

To validate this hypothesis, we overexpressed the full length and truncated (without the 5′ 1-303 bp) versions of *YALI1_C28456g*, and also deleted the full length and partial (5′ 0-33 bp) sequence of the gene (Fig. 3E). As shown in Fig. 3F, similar to the control strain, the full and partial gene deletion strains Xyl∆all and Xyl∆33 both grew poorly in the medium with xylose as the sole carbon source. In contrast, the full-length and truncated overexpression strains, Xylall and Xyl303, both showed improved growth compared to the control strain, especially when the *UT8* promoter was inserted into the 303-bp position of *YALI1_C28456*. This confirmed that the insertion of *UT8* promoter improved xylose metabolism by activating the expression of *YALI1_C28456*, not disrupting it. Based on these results, we concluded that amino acids 102-578 contain the functional domain of the protein that improves xylose metabolism (Fig. 3G).

### Determination of the pentose transport function of YALI1_C28456g

**Fig. 4:**
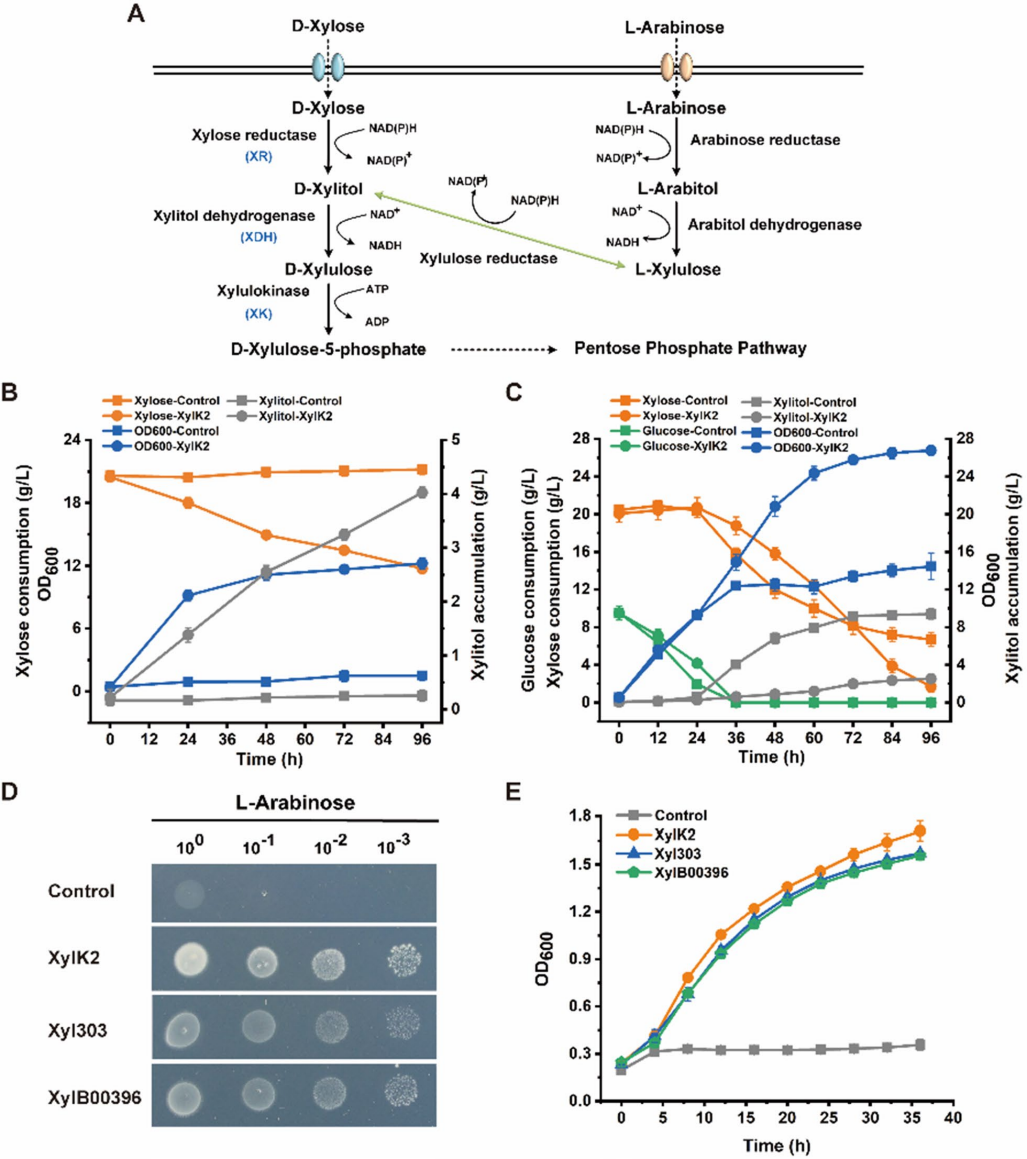
Verification of the pentose transport function of YALI1_C28456g. **A** Pentose metabolic pathway in *Y. lipolytica*. **B**, **C** Fermentation curves of the control strain *Y. lipolytica* Po1f and mutant strains with xylose as the sole carbon source **B** or glucose and xylose as dual carbon sources **C**. **D**, **E** Spot assay **D** and growth curves **E** of Po1f and the mutant strains with arabinose as the sole carbon source

Sequence alignment showed that YALI1_C28456g is a transporter protein. The transmembrane domain of YALI1_C28456g was predicted using an online tool TMHMM-2.0. There are 12 transmembrane helices, which are consistent with the 12 transmembrane helix domain of MFS proteins. In addition, YALI1_C28456g was fused with hrGFP for subcellular localization, and a strain expressing hrGFP alone was used as a control. As shown in Additional file 1: Figure S3A, hrGFP was mainly located in the cell membrane, confirming YALI1_C28456g was a membrane transporter. We also tested the *YALI1_C28456g* mutant strains for fermentation with xylose as the sole carbon source. As shown in Fig. 4B, the control strain did not consume xylose, while the mutant strain XylK2 could consume xylose without overexpression of the xylose pathway genes. The xylose consumption rate was 0.094 g/L/h and the optical density at 600 nm (OD_600_) reached 12, demonstrating that the activation of this gene promoted xylose utilization in *Y. lipolytica*. The accumulation of xylitol (0.44 g/g xylose) in strain XylK2 indicated that the expression of the endogenous xylose metabolism gene *ylXDH* was insufficient in *Y. lipolytica*. The transcription levels of endogenous xylose metabolism genes *ylXR*, *ylXDH* and *ylXK* in *Y. lipolytica* were determined, and all were found to be upregulated (Additional file 1: Figure S3B). Furthermore, we conducted glucose and xylose co-fermentation. As shown in Fig. 4C, compared with the control strain, the mutant strain XylK2 consumed more xylose and grew better. These results indicated that the activation of *YALI1_C28456g* may increase xylose transport and promote xylose metabolism in *Y. lipolytica*.

In addition to the xylose metabolism pathway, the arabinose metabolism pathway also exists in *Y. lipolytica*. The two pathways share some transporters and metabolic enzymes (Fig. 4A) [36]. Therefore, we tested the possibility that *YALI1_C28456g* could also promote arabinose transport. Both spot assays and growth characterization showed that the activation of *YALI1_C28456g* not only enhanced xylose metabolism, but also promoted growth using arabinose (Fig. 4D, E). Furthermore, we overexpressed a previously reported pentose transporter YALI0B00396p (YALI1_B00357g) [36] and observed similar improvements (Fig. 4D, E). It was demonstrated that the activation of *YALI1_C28456g* had a facilitative effect on both pentose metabolism.

The identified pentose transporter contributed to the utilization of xylose by *Y. lipolytica*, enabling the conversion of lignocellulose into high-value chemicals. Unlike *S. cerevisiae*, the regulation mechanism of pentose metabolism in *Y. lipolytica* remains unclear. Identification of key targets related to xylose metabolism would facilitate the cell factory construction and biorefining lignocellulose.

### Activation of YALI1_C28456g improves the production of β-farnesene from xylose in *Y. lipolytica*

**Fig. 5:**
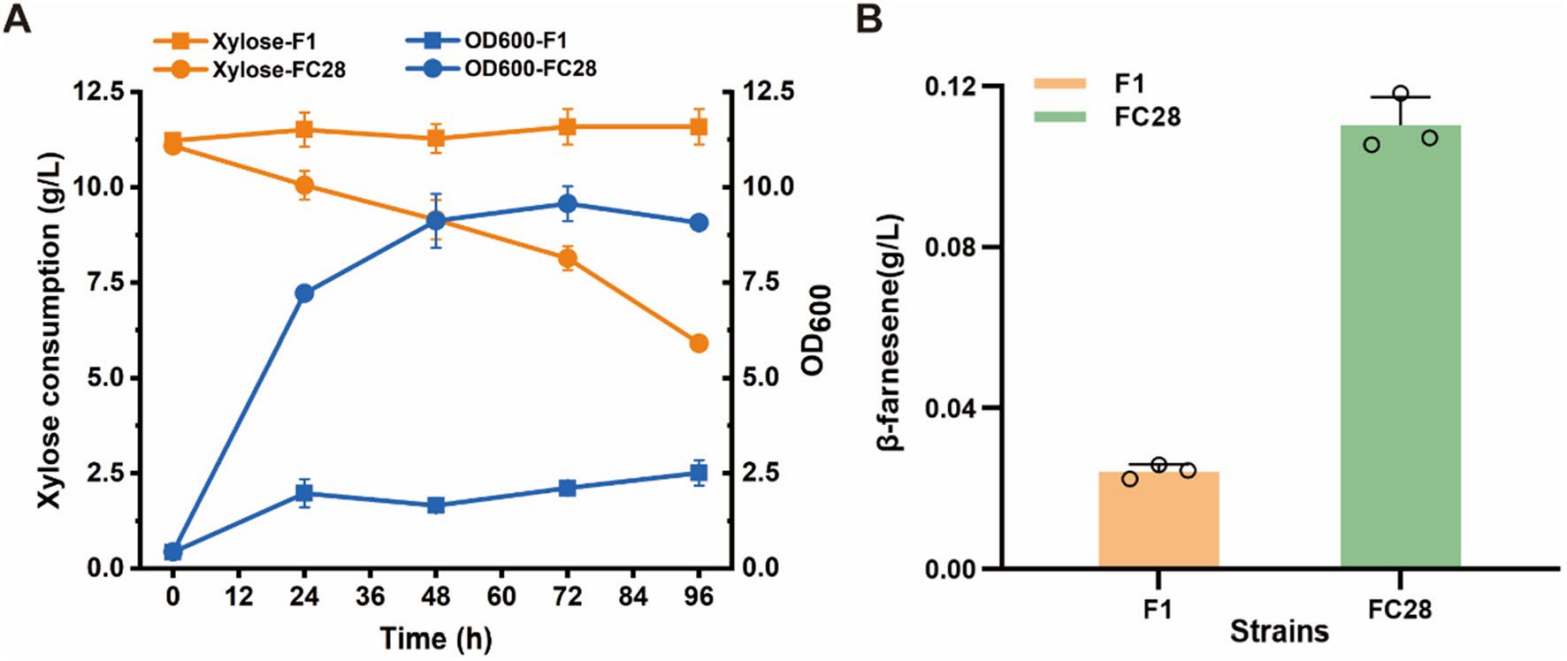
Fermentation characterization of the control and mutant strains of *Y. lipolytica* using shake-flask fermentation. **A** Substrate consumption and cell growth of the control strain F1 and the mutant strain with xylose as the sole carbon source. **B** The β-farnesene production of the control strain F1 and the mutant strain with xylose as the sole carbon source

β-farnesene (C_15_H_24_) is a non-cyclic volatile sesquiterpene produced by plants. It has shown great potential for market application in the fields of agriculture, industrial energy chemicals, medicine, health products and cosmetics [37, 38]. To explore whether the activation of *YALI1_C28456g* has a positive effect on the production of β-farnesene from xylose by *Y. lipolytica*, we used strain F1, which carries a β-farnesene synthase mutation (AanFS^K197T/F180H^) [39], and enhanced the mevalonate pathway. As expected, the control strain F1 did not consume xylose, and exhibited weak growth likely due to the utilization of amino acids or other trace amounts of available carbon sources in the YP medium. By contrast, the *YALI1_C28456g* overexpression strain FC28 consumed more xylose, and had a higher OD_600_ (Fig. 5A). As shown in Fig. 5B, the overexpression of *YALI1_C28456g* improved β-farnesene production from xylose. Engineered strain FC28 achieved a β-farnesene titer of 0.11 g/L, which was 4.4-fold higher than that of the control strain F1 (0.025 g/L). These results demonstrated the potential of *Y. lipolytica* to produce β-farnesene from xylose. Hence, further efforts to promote the conversion of xylose to β-farnesene through enhanced expression of xylose metabolism pathway genes and some pentose phosphate pathway genes should be considered.

## Conclusions

Genome-scale transcriptional regulation of genes is extremely important for cell factory construction and genetic analysis. In this study, we constructed NHEJ-mediated gene regulatory library and identify mutants that can activate gene expression. The activation mutants that improved acetic acid tolerance and activate pentose metabolism were rapidly screened. The NHEJ-mediated gene regulatory library provides a new approach to identify unknown targets related to specific phenotypes. This strategy can be easily applied to other microorganisms with high NHEJ efficiency, thereby facilitating large-scale genetic analysis.

## Material and methods

### Medium and culture conditions

*Escherichia coli* strain DH5α grown in Luria–Bertani broth supplemented with ampicillin (50 mg/L) at 37 °C was used for routine plasmid construction and subcloning. *Y. lipolytica* strains were cultivated at 30 °C in the following media: yeast extract peptone dextrose/xylose medium (YPD/YPX) containing 20 g/L glucose/xylose, 20 g/L tryptone and 10 g/L yeast extractor; or yeast nitrogen base medium (YNB) containing 20 g/L xylose, 1.7 g/L yeast nitrogen base and 5 g/L (NH_4_)_2_SO_4_ supplemented with suitable amino acid dropout mixes. Hygromycin B (600 mg/L) was added to the medium when necessary.

### Plasmid and strain construction

The strains and plasmids used in this study are listed in Additional file 1: Tables S3 and S4. The primers are listed in Additional file 1: Table S5. The plasmid YLEP-LEU [40] was digested with restriction enzymes NdeI and BstBI to obtain the *UT8* promoter fragment. The *LEU2* expression cassette and its backbone fragment were amplified from plasmid JMP114 [41] using primers UT8-LEU-F and UT8-LEU-R. Then the *UT8* promoter, *LEU2* expression cassette and its backbone fragments were assembled by Gibson to generate plasmid UT8-LEU-JMP. In addition, restriction enzyme sites SmaI and PmeI were added on both sides of the *LEU2-UT8* fragment to facilitate subsequent digestion of the plasmid UT8-LEU-JMP to obtain the *LEU2-UT8* integrated fragment.

The plasmid YLEP-LEU was digested with restriction enzymes NdeI and BstBI to obtain the *UT8* promoter fragment, *LEU2* expression cassette and its backbone fragment. The pDGA1-hrGFP fragment was amplified from plasmid B-3-pDGA1-hrGFP-CYC1t-LEU [42]. The *LEU2* expression cassette and its backbone fragment and pDGA1-hrGFP fragment were assembled by Gibson to generate plasmid 0-DGA1-hrGFP-YLEP-LEU. Then, the *UT8* promoter was assembled with the truncated *DGA1* promoter, leaving 50 bp, 250 bp, 500 bp, 750 bp or 1000 bp at the 3’end, respectively, and a series of plasmids 50-DGA1-hrGFP-YLEP-LEU, 250-DGA1-hrGFP-YLEP-LEU, 500-DGA1-hrGFP-YLEP-LEU, 750-DGA1-hrGFP-YLEP-LEU and 1000-DGA1-hrGFP-YLEP-LEU were generated by Gibson assembly. The above six plasmids were transformed into *Y. lipolytica* Po1f through lithium acetate transformation [43] for relative fluorescence detection.

### Design and construction of gene regulatory library

The *UT8* gene regulatory library was constructed with *Y. lipolytica* Po1f as the starting strain. The strong hybrid promoter *UT8* (*UAS1B8-TEF1*) and the *LEU2* expression cassette were used in tandem as an integration fragment, which was transformed into *Y. lipolytica* Po1f using a lithium acetate transformation method [43]. Transformants were cultured on SD-LEU selection plates at 30 °C for 2–3 days. Ultimately, approximately 2.0 × 10^5^ colonies were collected from 60 petri dishes (90 mm × 90 mm) to construct the gene regulation library.

### Screening of xylose metabolism-activated mutants in *Y. lipolytica*

The library mutants were inoculated into 5 mL YNB liquid medium with xylose (20 g/L) as the sole carbon source, and three independent test tubes were named K1, K2, and K3, respectively. In addition, the non-engineered strain Po1f was used as a control. After 6–7 days of cultivation, 200 μL of each culture was transferred to 5 mL fresh xylose medium and repeated once. Finally, appropriate amounts of each culture were spread on xylose plates to obtain colonies for subsequent growth characterization and insertion site mapping.

### Screening for acetic acid-tolerant mutants

To enrich for acetic acid-tolerant mutants, the gene regulation library mutants were screened by growth on YPD medium supplemented with an inhibitory concentration of acetic acid (70 mM). Mutants with improved growth were then selected for verification of acetic acid tolerance.

### Target identification and reverse engineering verification

Genomic DNA was extracted from the better-growing mutants for localization of insertion sites using enzymatic ligation-mediated intramolecular cyclization and Sanger sequencing [20]. The foreign *LEU2-UT8* DNA fragment was transformed into Po1f and inserted into the genome. The genomic DNA of the colonies was extracted and digested with DpnII (restriction site: 5’-GATC-3’). The digested DNA fragments were ligated and cyclized by T4 ligase, and the partial fragments of *LEU2-UT8* and associated genome sequences were amplified with specific outward-facing primers. Finally, PCR products were sequenced.

The integration fragments carrying the *LEU2-UT8* fragment and the 2 kb homologous arms were transformed into *Y. lipolytica* Po1f (*Δku70*) to obtain the targeted integration mutants.

### Growth curve characterization and spot assay

The strains were inoculated into 24-well plates containing 2 mL YPD medium and cultured at 30 °C overnight to obtain pre-cultures. Pre-culture precipitates were obtained by centrifugation, washed three times with sterile water and resuspended in 1 mL sterile water. Then they were transferred to fresh xylose/arabinose (20 g/L) medium at an inoculum volume of 2% (v/v), and grown in deep well plates at 220 rpm, 30 °C. The OD_600_ of cultures was detected using a Cytation microplate reader (BioTek).

In parallel, cell suspensions (OD_600_ = 1.0) were diluted in tenfold gradients, and 3-μL aliquots were dropped onto solid plates containing xylose/arabinose (20 g/L) as the sole carbon source and incubated at 30 °C for 3–5 days. Photographs were taken using an automatic colony counter. Three biological replicates were performed for each experiment.

### Multiple stress tolerance assays of NIP1 mutants

Pre-cultures of control strains and NIP1 mutant strains were incubated in 50 mL YPD medium with acetic acid (90 mM), NaCl (10%) or low pH (pH = 3) at 30 °C and 220 rpm in shaking flasks. Samples were taken every 12 h and subjected to growth curve characterization or spot assays. In addition, the strains were also analyzed for tolerance at high temperature (37 °C) conditions.

### Quantitative real-time PCR

Total RNA was purified using TRIzol reagent (Invitrogen). RNA was reverse transcribed to cDNA using a PrimeScript RT kit (Takara, China). qRT-PCR was performed in QuantStudioTM3 (ThermoFisher) in a total reaction volume of 30 μL per well. The housekeeping gene *ACT* was used as the internal reference. All assays were performed in triplicate, and a reaction without reverse transcriptase was used as a negative control.

### Subcellular localization

The plasmid encoding the C-terminus of the YALI1_C28456g transporter fused with *hrGFP* and the plasmid expressing only *hrGFP* were transferred into *Y. lipolytica* Po1f, respectively. The transformants were spread on SD-LEU selection plates to obtain monoclones, which were then inoculated into SD-LEU liquid medium. Cells cultured to mid-exponential phase were collected, and then resuspended in 50 mmol/L PBS buffer (pH = 6.5). Cells were spotted on microscope slides and images were acquired using an Olympus CX41 microscope equipped with a CCD camera (QIMAGING, Micropublisher 5.0 RTV) and mercury arc lamp (Olympus, URFLT50).

### Shake flask fermentation and sample analysis

Control strains and mutant strains were inoculated in 50 mL YP medium with xylose (10 g/L or 20 g/L) as the sole carbon source, or glucose (10 g/L) and xylose (20 g/L) as dual carbon sources, 220 rpm, 30 °C in shaking culture. Samples were taken every 24 h. OD_600_ values were measured using a spectrophotometer and used to plot growth curves. To collect β-farnesene specifically, 10% dodecane was added to the fermentation flasks during inoculation.

Fermentation samples were centrifuged to obtain supernatants. Xylose consumption, glucose consumption and xylitol production were detected by HPLC equipped with an Aminex HPX-87H column (BioRad, Inc., Hercules, CA) and a refractive index detector. The analysis was performed using 5 mM H_2_SO_4_ as the mobile phase at 0.6 mL/min, with a column temperature of 65 °C.

To detect the β-farnesene, the fermentation broth was centrifuged and the upper dodecane layer was collected for quantitative analysis by gas chromatography system equipped with a FID and an Rtx-5 capillary column, following a previously described [39].

### Supplementary Information

**Additional file1: Figure S1. **Evaluation of library capacity for mutants that can grow normally on hygromycin plates. (A) The growth of mutants with integrated *HYG* gene without promoter on hygromycin plate. (B) The growth of library mutants on hygromycin plate. (C) PCR confirmed that *HYG* gene expression was regulated by the inserted *UT8* promoter. **Figure S2. **Growth of control strain Po1f and library mutants in the medium with 70 mM acetic acid and the medium with xylose as the sole carbon source. (A, B) Growth of control strain Po1f (A) and library mutants (B) in the medium with 70 mM acetic acid. (C, D) Growth of control strain Po1f (C) and library mutants (D) in the medium containing xylose as the sole carbon source. **Figure S3.** Functional validation of YALI1_C28456g. (A) Subcellular localization based on GFP fluorescence of YALI1_C28456g. (B) Relative transcription levels of *ylXR*, *ylXDH* and *ylXK*. **Table S1.** Distribution of insertion sites of acetic acid-tolerant mutants. **Table S2.** Distribution of insertion sites of xylose metabolism-activated mutants. **Table S3.** The yeast strains used in this study. **Table S4.** The plasmids used in this study. **Table S5.** Sequences of the primers used in this study.

## Data Availability

The authors promise the availability of data and material.
