# Supplementary material for: Genome-scale transcriptional activation by non-homologous end joining-mediated integration in Yarrowia lipolytica
Source: Biotechnol Biofuels Bioprod. 2024 Feb 15;17:24. doi: 10.1186/s13068-024-02472-x (PMC10870441; doi:10.1186/s13068-024-02472-x)
Supplement: Supplementary file 1 — Additional file1: Figure S1. Evaluation of library capacity for mutants that can grow normally on hygromycin plates. (A) The growth of mutants with integrated HYG gene without promoter on hygromycin plate. (B) The growth of library mutants on hygromycin plate. (C) PCR confirmed that HYG gene expression was regulated by the inserted UT8 promoter. Figure S2. Growth of control strain Po1f and library mutants in the medium with 70 mM acetic acid and the medium with xylose as the sole carbon source. (A, B) Growth of control strain Po1f (A) and library mutants (B) in the medium with 70 mM acetic acid. (C, D) Growth of control strain Po1f (C) and library mutants (D) in the medium containing xylose as the sole carbon source. Figure S3. Functional validation of YALI1_C28456g. (A) Subcellular localization based on GFP fluorescence of YALI1_C28456g. (B) Relative transcription levels of ylXR, ylXDH and ylXK. Table S1. Distribution of insertion sites of acetic acid-tolerant mutants. Table S2. Distribution of insertion sites of xylose metabolism-activated mutants. Table S3. The yeast strains used in this study. Table S4. The plasmids used in this study. Table S5. Sequences of the primers used in this study. [file 13068_2024_2472_MOESM1_ESM.docx]

**Additional File Information**

**Genome-scale transcriptional activation by non-homologous end joining-mediated integration** **in *Yarrowia lipolytica***

Xiaoqin Liu^a^, Jingyu Deng^a^, Jinhong Zhang^a^, Zhiyong Cui^a^, Qingsheng Qi^a,^*, Jin Hou^a,^*

^a^State Key Laboratory of Microbial Technology, Shandong University, 266237 Qingdao, P. R. China.

*To whom correspondence should be addressed:

Qingsheng Qi, State Key Laboratory of Microbial Technology, Shandong University, Binhai Road 72, Qingdao, Shandong, 266237, P. R. China; Tel: +86 532 58632580; Email: qiqingsheng@sdu.edu.cn.

Jin Hou, State Key Laboratory of Microbial Technology, Shandong University, Binhai Road 72, Qingdao, Shandong, 266237, P. R. China; Tel: +86 532 58632401; Email: houjin@sdu.edu.cn.

**
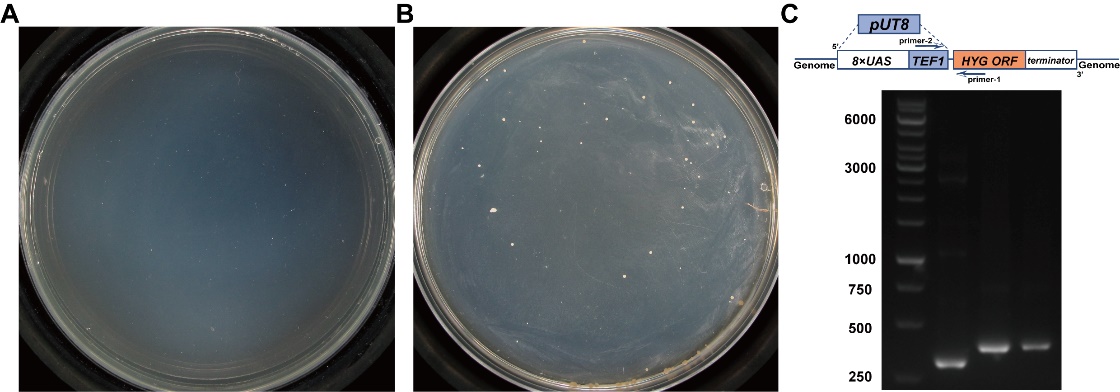
**

**Additional file 1: Figure S1.** Evaluation of library capacity for mutants that can grow normally on hygromycin plates. (A) The growth of mutants with integrated *HYG* gene without promoter on hygromycin plate. (B) The growth of library mutants on hygromycin plate. (C) PCR confirmed that *HYG* gene expression was regulated by the inserted *UT8* promoter. We designed primers at the upstream of the *HYG* gene and the downstream of the *UT8* promoter for PCR amplification, and obtained two different transformers.

**
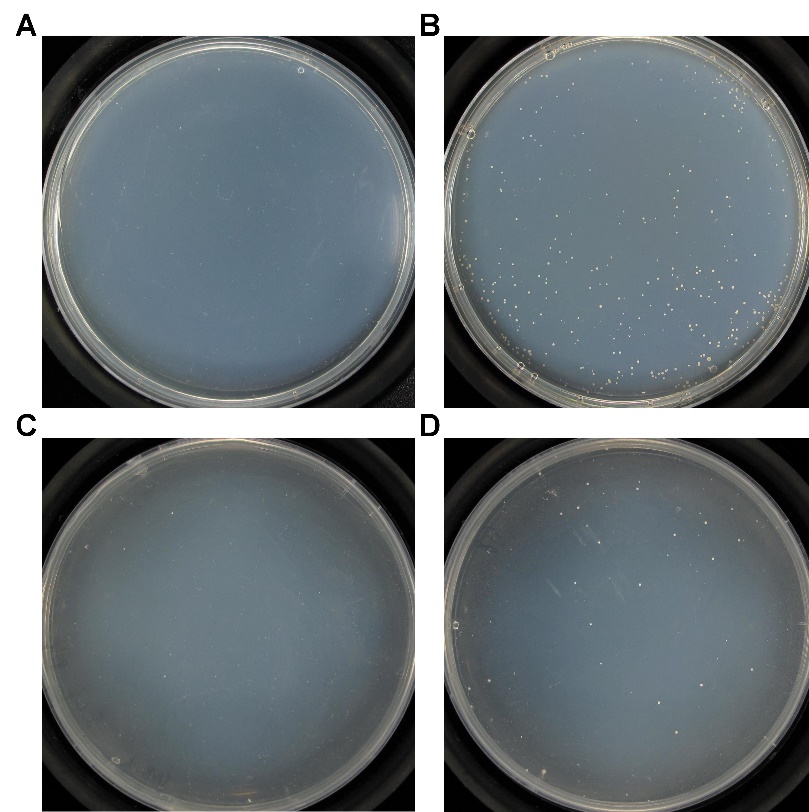
**

**Additional file 1: Figure S2.** Growth of control strain Po1f and library mutants in the medium with 70 mM acetic acid and the medium with xylose as the sole carbon source. (A, B) Growth of control strain Po1f (A) and library mutants (B) in the medium with 70 mM acetic acid. (C, D) Growth of control strain Po1f (C) and library mutants (D) in the medium containing xylose as the sole carbon source.


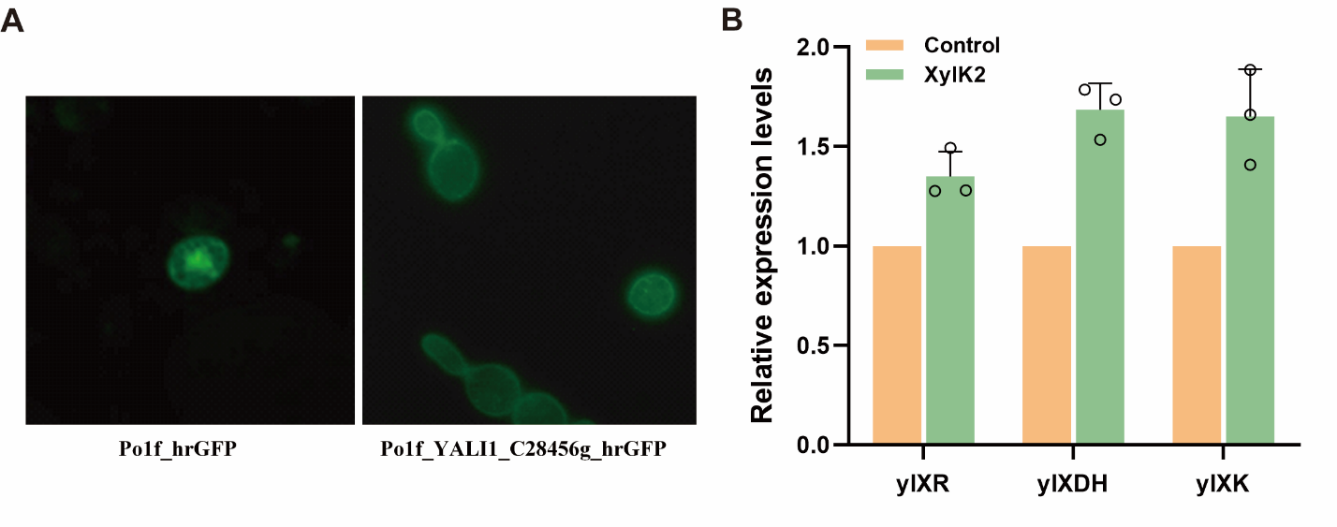


**Additional file 1:** **Figure S3.** Functional validation of YALI1_C28456g. (A) Subcellular localization based on GFP fluorescence of YALI1_C28456g. (B) Relative transcription levels of *ylXR*, *ylXDH* and *ylXK*.

**Additional file 1:** **Table S1.** Distribution of insertion sites of acetic acid-tolerant mutants.

| Mutants | Insertion position | Function annotation |
| --- | --- | --- |
| 3 | 276 bp upstream of *YALI1_B16477g* coding region. | Homologous to *VAN1* of *Saccharomyces cerevisiae*, component of the mannan polymerase I. |
| 6 | 4 bp upstream of *YALI1_D04331g* coding region. | RNA polymerase I-associated factor. |
| 4/8/10/11 | 735 bp upstream of *YALI1_D28825g* coding region. | Homologous to *NIP1* of *Saccharomyces cerevisiae*. |

**Additional file 1:** **Table S2.** Distribution of insertion sites of xylose metabolism-activated mutants.

| Mutants | Insertion position | Function annotation |
| --- | --- | --- |
| K1-6 | 34 bp upstream of *YALI1_C28456g* coding region. | The major facilitator superfamily (MFS) structural domain protein. |
| K1-18 |  |  |
| K2-1 | 33 bp upstream of *YALI1_C28456g* coding region. |  |
| K2-2 |  |  |
| K2-20 |  |  |
| K3-13 |  |  |
| K3-14 | 37 bp upstream of *YALI1_C28456g* coding region. |  |
| K3-18 |  |  |

**Additional file 1: Table S3.** The yeast strains used in this study.

| Strains | Description | References |
| --- | --- | --- |
| Po1f | *MatA*, *Leu2-270*, *URA3-302*, *xpr2-322*, *axp-2* | INRA |
| Po1f-hyg | Random integration of the *hyg* gene without promoter in the Po1f strain | This stduy |
| Po1f-hyg-*LEU2*-*UT8* | Po1f-hyg strain with *LEU2-UT8* random genome integration | This stduy |
| NIP1-735 | Integrate *LEU2-UT8* at 735 bp upstream of *NIP1* coding region, Po1f | This stduy |
| R-NIP1 | Targeted integration of *LEU2-UT8* at 735 bp upstream of *NIP1* coding region, Po1f (*∆ku70*) | This stduy |
| Po1f-*LEU2*-*UT8* | Po1f strain with *LEU2-UT8* random genome integration | This stduy |
| Po1f (*∆ku70*) | Po1f strain with *ku70* knockout | This stduy |
| Po1f (*∆ku70*)-*LEU2* | Po1f strain with *ku70* knockout and *LEU2* marker random genome integration | This study |
| XylK2 | Integrate *LEU2-UT8* at 33 bp upstream of *YALI1_C28456g* coding region, Po1f | This study |
| Xyl33 | Targeted integration of *LEU2-UT8* at 33 bp upstream of *YALI1_C28456g* coding region, Po1f (*∆ku70*) | This study |
| Xylall | Targeted integration of *UT8-YALI1_C28456g-CYC1t-LEU2*, Po1f (*∆ku70*) | This study |
| Xyl303 | Targeted integration of *UT8-303YALI1_C28456g-CYC1t-LEU2*, Po1f (*∆ku70*) | This study |
| Xyl∆all | *ΔYALI1_C28456g* :: *LEU2,* Po1f (*∆ku70*) | This study |
| Xyl∆33 | *Δ*33 bp upstream of the YALI1_C28456g coding region :: *LEU2*, Po1f (*∆ku70*) | This study |
| XylB00396 | Po1f strain with *UT8-YALI0B00396p-CYC1t* random genome integration | This study |
| F1 | Po1f strain that expressed a β-farnesene synthase mutant (AanFS^K197T/F180H^) and enhanced the mevalonate pathway | This study |
| FC28 | F1 strain with *UT8-303YALI1_C28456g-CYC1t* random genome integration | This study |

**Additional file 1: Table S4.** The plasmids used in this study.

| Plasmids | Description | References |
| --- | --- | --- |
| JMP-hyg | hyg marker flanking with lox sites | [1] |
| YLEP-LEU | Episomal vector with *LEU2* marker, *UT8* promoter and *CYC1* terminator | [2] |
| B-3-pDGA1-hrGFP-CYC1t-LEU | B-3 chromosomal integration site, episomal vector with *LEU2* marker,1kb homology arms and *pDGA1-hrGFP-CYC1t* expression cassette | [3] |
| 0-DGA1-hrGFP-YLEP-LEU | Episomal vector with *LEU2* marker and *pDGA1-hrGFP-CYC1t* expression cassette | This study |
| 50-DGA1-hrGFP-YLEP-LEU | Episomal vector with *LEU2* marker and *pDGA1-hrGFP-CYC1t* expression cassette, insert *pUT8* at 50 bp of *pDGA1* | This study |
| 250-DGA1-hrGFP-YLEP-LEU | Episomal vector with *LEU2* marker and *pDGA1-hrGFP-CYC1t* expression cassette, insert *pUT8* at 250 bp of *pDGA1* | This study |
| 500-DGA1-hrGFP-YLEP-LEU | Episomal vector with *LEU2* marker and *pDGA1-hrGFP-CYC1t* expression cassette, insert *pUT8* at 500 bp of *pDGA1* | This study |
| 750-DGA1-hrGFP-YLEP-LEU | Episomal vector with *LEU2* marker and *pDGA1-hrGFP-CYC1t* expression cassette, insert *pUT8* at 750 bp of *pDGA1* | This study |
| 1000-DGA1-hrGFP-YLEP-LEU | Episomal vector with *LEU2* marker and *pDGA1-hrGFP-CYC1t* expression cassette, insert *pUT8* at 1000 bp of *pDGA1* | This study |
| JMP114 | Integrative vector with *LEU2* marker flanked by Lox sites | [2] |
| UT8-LEU-JMP | Integrative vector with *LEU2* marker and *UT8* promoter | This study |
| UT8-LEU-NIP1 | Integrative vector with *LEU2* marker and *pUT8-NIP1-CYC1t* expression cassette | This study |
| UT8-LEU-33C28456 | Integrative vector with *LEU2-UT8* fragment and flanking homology arm sequences | This study |
| UT8-303C28456 hrGFP-LEU | Episomal vector with *LEU2* marker and *pUT8-303YALI1_C28456g hrGFP-CYC1t* expression cassette | This study |
| UT8-C28456-JMP | Integrative vector with *LEU2* marker and *pUT8-YALI1_C28456g-CYC1t* expression cassette | This study |
| UT8-303C28456-JMP | Integrative vector with *LEU2* marker and *pUT8-303YALI1_C28456g-CYC1t* expression cassette | This study |
| ∆C28456-JMP | Integrative vector with *LEU2* marker flanked by Lox sites and homology arm sequences | This study |
| ∆33-JMP | Integrative vector with *LEU2* marker flanked by Lox sites and homology arm sequences | This study |
| UT8-B00396-JMP | Integrative vector with *LEU2* marker and *UT8-B00396-CYC1t* expression cassette | This study |

**Additional file 1: Table S5.** Sequences of the primers used in this study.

| Name | Sequence (5’-3’) | Description |
| --- | --- | --- |
| yli-hyg-F | ATGAAAAAGCCTGAACTCACC | Construction of *hyg* integration fragment |
| yli-hyg-R | AATTACCCTGTTATCCCTAGCCCTTTTTATAGAGTCTTATACAC | Construction of *hyg* integration fragment |
| YLEP-F | TCATGTAATTAGTTATGTCACGCTTACATTCACGC | DGA1-related plasmid construction |
| YLEP-R | GCGTTTTTCCATAGGCTCCGCCCCC | DGA1-related plasmid construction |
| hrGFP-DGA1-F | CAAGTCACACAAAACAAAAGCTATTTAAATATGGTGAGCAAGCAGATCCTGAAG | DGA1-related plasmid construction |
| hrGFP-DGA1-R | GTGAATGTAAGCGTGACATAACTAATTACATGATTACACCCACTCGTGCAGGCTGCCC | DGA1-related plasmid construction |
| DGA1-F | GGGGGCGGAGCCTATGGAAAAACGCCATATGGTAGCACCAGTTGCAAGTGGGATTCG | DGA1-related plasmid construction |
| DGA1-R | CACCATATTTAAATAGCTTTTGTTTTGTGTGACTTGTCTG | DGA1-related plasmid construction |
| 50-DGA1-F | CCTTCTGAGTATAAGAATCATTCAAAGACTTTTTCTTCTAACAACAGGCAACAGAC | DGA1-related plasmid construction |
| 50-DGA1-R | GGTACCGCATGCTTCCTTGGTACCTTGGGAGCTTATCAGTCACGGTCCAC | DGA1-related plasmid construction |
| 250-DGA1-F | CCTTCTGAGTATAAGAATCATTCAAAGACTCCTCTACACACGTCAAATCCG | DGA1-related plasmid construction |
| 250-DGA1-R | GGGTACCGCATGCTTCCTTGGTACCTTCGGTTTCGAGGAGCAAGGAGGAC | DGA1-related plasmid construction |
| 500-DGA1-F | CATTTCCTTCTGAGTATAAGAATCATTCAAAGCACGCCCCCGAAACTCTTTCTCC | DGA1-related plasmid construction |
| 500-DGA1-R | AATTCGGGTACCGCATGCTTCCTTGGTACCTTGTCTGCAGAGAGAAGCCCGGTTTG | DGA1-related plasmid construction |
| 750-DGA1-F | TTTCCTTCTGAGTATAAGAATCATTCAAAGTTTCGTCTATCCTGAACTGAGTTTTTTTC | DGA1-related plasmid construction |
| 750-DGA1-R | GAATTCGGGTACCGCATGCTTCCTTGGTACCAGAGAAAAGCGAACCATGGGGTTG | DGA1-related plasmid construction |
| 1000-DGA1-F | TTCCTTCTGAGTATAAGAATCATTCAAAGATGCTGCGGGCGGATCCTGGTGCATTTTTG | DGA1-related plasmid construction |
| 1000-DGA1-R | AATTCGGGTACCGCATGCTTCCTTGGTACCAAAAGAGGTGTCCGATATTTTCGAATCCC | DGA1-related plasmid construction |
| UT8-LEU-F | GGGTACCGCATGCTTCCTTGGTACCTTCATAACGCGTGTACGCATGTAACATTATACTG | UT8-LEU-JMP plasmid construction |
| UT8-LEU-R | CCTTCTGAGTATAAGAATCATTCAAAGGTTTGTGGAGCTCCAATCGCCCTATAGTG | UT8-LEU-JMP plasmid construction |
| 33C28456-UP-F | UCATTAATGCAGCTGGCACGACAAGCGGCCGCGTTTAGGAGCACAGTTGACTTTTCTG | UT8-LEU-33  C28456 construction |
| 33C28456-UP-R | CCTGGCGTTACCCAACTTAATCGCCTGTTTCTGCAGCTGCAGATGTGGGTAAG | UT8-LEU-33  C28456 construction |
| 33C28456-Down-F | GGTGGTAGCTTGTTACTGTATATTCGACCCATATGACGCACAATTATGCAGCCG | UT8-LEU-33  C28456 construction |
| 33C28456-Down-R | CGCTATTACGCCAGCTGTTGTTTAAACTGCTTAGTAATGGTATGTCGTTAATGATT | UT8-LEU-33  C28456 construction |
| Xyl-F | GTTTAAACAACAGCTGGCGTAATAGCGAAGAG | UT8-LEU-33  C28456 construction |
| Xyl-R | GCGGCCGCTTGTCGTGCCAGCTGCATTAATG | UT8-LEU-33  C28456 construction |
| 303C28456-F | ATGAATCCCATTGAATTGGAGCGCAT | UT8-303C28456 hrGFP-LEU construction |
| 303C28456-R | CTGCTTGCTCACCATCTGTCCGTGGGTACCTCTCTGGTAG | UT8-303C28456 hrGFP-LEU construction |
| Primer-1 | AAGCACTTCCGGAATCGGGAG | UT8-HYG validation |
| Primer-2 | CGTCCCCGAATTACCTTTCCTCTTC | UT8-HYG validation |

Supplementary References

1. Cui ZY, Jiang X, Zheng HH, Qi QS, Hou J: Homology-independent genome integration enables rapid library construction for enzyme expression and pathway optimization in *Yarrowia lipolytica*. *Biotechnol Bioeng* 2019, 116(2):354-363. 10.1002/bit.26863.

2. Cui Z, Zheng H, Zhang J, Jiang Z, Zhu Z, Liu X, Qi Q, Hou J: A CRISPR/Cas9-Mediated, Homology-Independent Tool Developed for Targeted Genome Integration in *Yarrowia lipolytica*. *Appl Environ Microbiol* 2021, 87(6)10.1128/AEM.02666-20.

3. Liu X, Cui Z, Su T, Lu X, Hou J, Qi Q: Identification of genome integration sites for developing a CRISPR-based gene expression toolkit in *Yarrowia lipolytica*. *Microb Biotechnol* 2022, 15(8):2223-2234. 10.1111/1751-7915.14060.
